# How Can Vaccines Against Influenza and Other Viral Diseases Be Made More Effective?

**DOI:** 10.1371/journal.pbio.1000571

**Published:** 2010-12-21

**Authors:** Peter L. Nara, Gregory J. Tobin, A. Ray Chaudhuri, Jessie D. Trujillo, George Lin, Michael W. Cho, Simon A. Levin, Wilfred Ndifon, Ned S. Wingreen

**Affiliations:** 1 Biological Mimetics, Inc., Frederick, Maryland, United States of America; 2 Department of Biomedical Sciences, College of Veterinary Medicine, Iowa State University, Ames, Iowa, United States of America; 3 Department of Veterinary Preventive Medicine and Microbiology, Iowa State University, Ames, Iowa, United States of America; 4 Department of Ecology and Evolutionary Biology, Princeton University, Princeton, New Jersey, United States of America; 5 Department of Immunology, The Weizmann Institute of Science, Rehovot, Israel; 6 Department of Molecular Biology, Princeton University, Princeton, New Jersey, United States of America

A large fraction of the world's most widespread and problematic pathogens, such as the influenza virus, seem to persist in nature by evading host immune responses by inducing immunity to genetically and phenotypically plastic epitopes (aka antigenic variation). The more recent re-emergence of pandemic influenza A/H1N1 and avian H5N1 viruses has called attention to the urgent need for more effective influenza vaccines. Developing such vaccines will require more than just moving from an egg-based to a tissue-culture–based manufacturing process. It will also require a new conceptual understanding of pathogen–host interactions, as well as new approaches and technologies to circumvent immune evasion by pathogens capable of more genetic variation. Here, we discuss these challenges, focusing on some potentially fruitful directions for future research.

Two important challenges for current influenza research are to explain the mechanisms involved in creating and maintaining the highly restricted diversity of epidemic strains and to develop more broadly efficacious vaccines capable of protecting against future epidemics. The continued epidemiological importance of the influenza virus derives in part from its ability to generate new annual strains capable of evading host immunity. This plasticity is generally thought to occur mostly through a combination of random genetic mutations, associated with an error-prone polymerase, and genetic reassortment. We argue here that the observed strain-to-strain, year-to-year variation is in part a consequence of another important contributor to the rapid emergence of immune-evading variants, namely the propensity of the host immune system to develop antibodies to immunodominant epitopes (i.e., epitopes for which there is a preferred immune response by the host) located in variable regions of the viral envelope protein(s) (e.g., HA and NA). The interesting and paradoxical outcome of this immunodominant epitope–antibody interaction is that it appears to lead to effective, highly strain-specific antibodies while at the same time (due partly to the proximity of these epitopes to the conserved cell-receptor binding site found on the viral envelope) sterically interfering with the generation of more broadly reactive antibodies [1]–[4]. The virus's ability to mutate, together with other host, ecological, and other evolutionary factors, still provide a chicken-and-egg puzzle. It is not yet well understood how these factors combine to produce the characteristic patterns of influenza epidemiology, including seasonality in the northern and southern hemispheres, apparent endemicity in the tropics, and a single-trunk phylogeny for the proteins (viral envelope-HA and surface neuraminidase-NA) most often targeted by antibodies [5]–[6]. This latter fact implies that a very limited number of distinct strains are responsible for epidemics at any given time.

Thus far, several possible explanations have been proposed for the very limited diversity of epidemic strains (see Box 1): that mutations occurring along one dimension of a presumed two-dimensional “strain space” may be intrinsically deleterious [7], that the viral infection produces a short-lived strain-transcending immunity [6], or that the virus may be evolving on a phenotypically neutral network [8]. Additional insight will likely come from models that integrate some of the features discussed in this essay and essential features of the virus's phenotype (particularly its high mutability and its tendency to form genetic clusters that are potential targets of natural selection [9]), the host immune response (particularly its propensity to target variable epitopes that have differing abilities to support viral neutralization [1]–[2],[4]), and host ecology to predict the virus's phylogeny and evolution.

**Box 1. What Limits the Diversity of Epidemic Strains?**

In spite of the very high viral mutation rates, the phylogenies of the proteins that appear to be evolving under the highest degree of immune selection pressure (such as the HA1 protein of H3N2 influenza virus), as measured by the ratio of nonsynonymous to synonymous nucleotide changes occurring at known epitopic sites, have only a single trunk, implying a very limited genetic diversity of those proteins and, hence, of epidemic strains, and many short branches. Here, we highlight three proposed explanations for this peculiar phylogenetic structure

- **Low effective dimensionality of the space of viral phenotypes**
- Suppose, for simplicity, that the features of the viral phenotype most important for its spread among hosts are its transmissibility and the epitopes most readily recognized by the immune system. If the effects of immune recognition of different epitopes are not independent (e.g., due to interference among antibodies to those epitopes), then the number of effective epitopes (and, hence, the effective dimensionality of the component of phenotype space represented by those epitopes) would be smaller than the total number of epitopes. Further, if (i) there are only two effective epitopes and (ii) for a particular viral genetic background and structure of host immunity, mutations to one of those epitopes (denoted epitope X) decrease viral transmissibility, then mutations that give rise to epidemic strains would mostly occur in the other epitope. The virus's phylogeny would therefore contain a single trunk representing the lineage of the epidemic strains [7]. Nevertheless, there may be a limited spread of strains carrying mutations to epitope X, which would occur on the branches of the phylogeny, once host immunity renders the normally more transmissible strains less able to spread.
- **Degeneracy of the mapping from genotypes to phenotypes**
- Suppose that most mutations to a particular effective epitope have a negligible effect on immune recognition of that epitope. Such mutations would produce a “neutral” network of epitope genotypes having similar immunological phenotypes. During evolution the virus population would sample the neutral networks associated with the effective epitopes while searching for genotypes whose phenotypes are less recognizable to the immune system. This would cause the virus's genetic diversity to increase. If an immunologically novel phenotype is found, then, depending on the selection pressure and the fitness differential between that phenotype and the virus population's modal phenotype, the virus would start sampling the neutral network of the new genotype. The ensuing selective sweep will weed out the previously sampled genotypes, reducing the virus's genetic diversity—only the lineage of the particular genotype that “found” the fitter genotype would survive. Over time, both the repeated sampling of phenotypically neutral networks of genotypes and the occasional selective sweeps would produce a single-trunk viral phylogeny containing many short-lived side branches [8].
- **Strain-transcending immunity**
- Suppose that infection with one influenza virus subtype induces partial immunity against all subtypes with a half-life sufficient to largely prevent the possibility of reinfection within the same influenza season. Such a heterotypic immunity would contribute to a high rate of strain turnover and a limited diversity of co-circulating influenza strains during inter-pandemic periods. In addition, during pandemic events heterotypic immunity might cause the elimination of an existing subtype by a new (pandemic) subtype, depending on the level of pre-existing host immunity to the latter [6],[10].

The ability to predict accurately the influenza virus phylogeny and evolution would enable the prediction of imminent circulating strains and assist in the development of more effective conventional vaccines, which primarily target variable epitopes of the viral coat proteins. To be effective, past and current influenza vaccines require a close match between the corresponding epitopes of the vaccine strain and those of the rapidly evolving, circulating strains, an objective that is often difficult to achieve. Vaccines that target more conserved functional epitopes would further improve on conventional vaccines by protecting against a wider range of strains and being effective for a longer duration [11]. There are ongoing efforts to design such vaccines, some of which have already led to vaccine candidates that are undergoing clinical trials [12]. In order to achieve optimal breadth of coverage with this new generation of vaccines there is a continued need for basic research to elucidate the reasons underlying the lack of or poor immunogenicity of more conserved functional domains located next to immunodominant variable epitopes, to explain the reduced plasticity of these conserved epitopes, and to predict the potential impact of sustained immune pressure on these conserved targets.

Likely to prove central to the successful design of a new generation of vaccines is a more nuanced understanding of pathogen–host interactions. In particular, two mechanisms of immune evasion—deceptive imprinting and antibody interference—deserve greater attention. Deceptive imprinting, originally described as clonal dominance [1]–[2] and later expanded to include a more complex interaction of immunodominance coupled to antigenic variation [3],[13]–[15], posits that pathogens, such as influenza, have evolved epitopes that combine immunodominance, antigenic/genetic variation, and other poorly understood mechanisms involving immune regulation to decrease the effectiveness of immune responses (both of antibodies and T cells) to infection/vaccination, allowing viral escape from immune surveillance (see Box 2).

**Box 2. Deceptive Imprinting**

The host adaptive immune system has evolved to recognize and respond preferentially to biochemical structures which are deemed “foreign” or “non-self.” How “foreignness” is ultimately determined by the host immune system continues to be a major question of immunology and takes on even more importance with the models proposed in this perspective. Immunodominance—defined as a heightened and preferred immune response by the host to a limited set of epitopes—was originally described in and thought to be purely a phenomenon of the major histocompatibility complex (MHC)–restricted response genes in inbred mouse strains. Although experimental immunologists noted pronounced or unusually strong antibody responses in certain host–antigen interactions, the idea of immunodominance was not readily applied to host–pathogen interactions (reviewed in [16] and [17]). The importance of the theory of “deceptive imprinting” is that it advances certain aspects of an earlier described phenomenon called “original antigenic sin” [18] (also known as the Hoskins effect) by implying that immunodominance is the driving mechanism that operates and is selected for in pathogens in a genetically outbred host setting.

Once immunodominance of epitopes is exploited by pathogens and placed into an immune memory of the host, immunodominance leads to the propensity of the immune system to preferentially utilize immunological memory based on a previous infection by a foreign entity (e.g., a virus, bacterium, or parasite) when a second slightly different version of that entity is encountered. This leaves the immune system somewhat “trapped” by the first response it has made to each entity, and unable to mount potentially more effective responses during subsequent infections. Thus, if pathogens couple cross-reactive immunodominance (hetero-specific immunity) to strain-specific immunity and evolve these immunodominant epitopes structurally next to highly conserved functional domains needed by the virus to infect host cells, the pathogens would elicit a host immune response that is mostly directed to less protective epitopes—hence deceptive imprinting.

**Figure 1:**
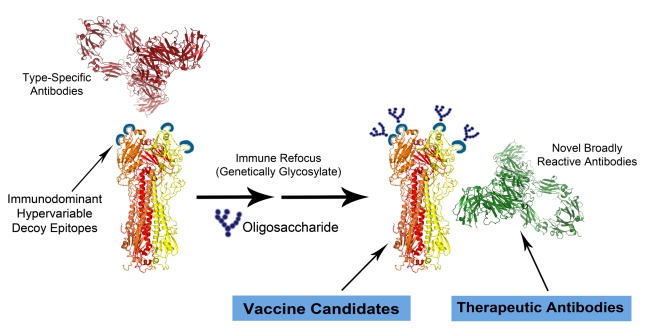
Diagram of immune refocusing technology and steric antibody interference using influenza hemagglutinin (HA) trimer as an example (modified from [**3**]). The molecular structures in the figure are drawn to scale to demonstrate the relative sizes of the reactants. In the left panel, native HA containing immunodominant decoy epitopes induce type-specific antibodies shown in red. In the middle panel, the HA has been engineered to include additional N-linked glycans at specific sites in the epitopes (alternatively, point mutations or deletions can be engineered into these sites). In the right panel, the immune refocused HA antigen elicits broadly reactive immune responses (shown as a green antibody) and can be used as a vaccine or to derive novel therapeutic antibodies having broad reactivity. The figure also highlights the potential impact of antibody interference because the width of the distal surface of each Fab fragment of an antibody is comparable to the diameter of a native HA trimer, antibodies that bind to different sites in the globular head of trimeric HA can sterically interfere with each other as previously shown (e.g., [21]). The combination of deceptive imprinting and steric interference can produce oligoclonal, rather than polyclonal, immune responses that are largely skewed towards the most immunodominant and variable epitopes in the pathogen.

This insight suggests that by molecularly modifying immunodominant epitopes in such a way as to immune-dampen them (called, “immune refocusing”; see Figure 1) as part of a vaccine strategy, it may be possible to recruit the B and T cell repertoires of vaccine-induced antibodies away from “pathogen-evolved” immunodominant deceptive epitopes to more conserved epitopes, and thereby to potentially sharply increase vaccine efficacy. The feasibility of this approach to refocusing the immune response is supported by initial experimental studies on HIV-1 and influenza virus [3],[13],[19],[20].

In the case of B cell immunity to influenza, antibody interference can contribute to deceptive imprinting. Specifically, antibodies to epitopes with either zero or low neutralization efficiencies (highly type or strain-restricted) may sterically interfere with antibodies to epitopes with high neutralization efficiencies [4]. These low-efficiency epitopes need not always be immunodominant to significantly decrease overall viral neutralization, as long as their immunological sub-dominance is offset by a compensating higher affinity of their cognate antibodies. Essentially, because of steric hindrance, antibodies are in a competition to bind to viral coat proteins, and the antibodies with the highest ratio of free concentration to binding constant will win, even if those antibodies are inefficient at neutralizing the virus. In addition to decreasing viral neutralization, the interference provided by low-efficiency antibodies can also decrease the extent to which a virus must mutate its high-efficiency epitopes in order to infect a vaccinated host or reinfect a previously infected host. However, by identifying and genetically modifying the low-efficiency epitopes used in vaccines, it may be possible to decrease such antibody interference and thereby greatly improve viral neutralization by vaccine-induced high-neutralization efficiency antibodies [4].

Vaccines often take between 16 and 20 years to develop, and the challenge now is to understand deceptive imprinting better and to systematically identify and characterize deceptive epitopes and low-efficiency, interfering epitopes [3]–[4] in influenza and other viruses. Progress here would enable targeting of both immunodominant deceptive epitopes and low-efficiency epitopes for genetic modification. In addition, more studies are needed to determine whether such genetic modifications can actually lead to significantly greater vaccine efficacy (e.g., [3],[19]), but there is great promise in these understanding-driven approaches.
